# Fostering interdisciplinary collaboration: A longitudinal social network analysis of the NIH mHealth Training Institutes

**DOI:** 10.1017/cts.2021.859

**Published:** 2021-09-20

**Authors:** Eric Ho, Minjeong Jeon, Minho Lee, Jinwen Luo, Angela F Pfammatter, Vivek Shetty, Bonnie Spring

**Affiliations:** 1 Department of Education, UCLA, Los Angeles, California, USA; 2 Division of Diagnostic and Surgical Sciences, School of Dentistry, UCLA, Los Angeles, California, USA; 3 Department of Preventive Medicine, Northwestern University Feinberg School of Medicine, Chicago, Illinois, USA

**Keywords:** Team science, mHTI, program evaluation, longitudinal network analysis, communications, team homophily, gender homophily

## Abstract

**Background/Objective:**

Growing recognition that collaboration among scientists from diverse disciplines fosters the emergence of solutions to complex scientific problems has spurred initiatives to train researchers to collaborate in interdisciplinary teams. Evaluations of collaboration patterns in these initiatives have tended to be cross-sectional, rather than clarifying temporal changes in collaborative dynamics. Mobile health (mHealth), the science of using mobile, wireless devices to improve health outcomes, is a field whose advancement needs interdisciplinary collaboration. The NIH-supported annual mHealth Training Institute (mHTI) was developed to meet that need and provides a unique testbed.

**Methods:**

In this study, we applied a longitudinal social network analysis technique to evaluate how well the program fostered communication among the disciplinarily diverse scholars participating in the 2017−2019 mHTIs. By applying separable temporal exponential random graph models, we investigated the formation and persistence of project-based and fun conversations during the mHTIs.

**Results:**

We found that conversations between scholars of different disciplines were just as likely as conversations within disciplines to form or persist in the 2018 and 2019 mHTI, suggesting that the mHTI achieved its goal of fostering interdisciplinary conversations and could be a model for other team science initiatives; this finding is also true for scholars from different career stages. The presence of team and gender homophily effects in certain years suggested that scholars tended to communicate within the same team or gender.

**Conclusion:**

Our results demonstrate the usefulness of longitudinal network models in evaluating team science initiatives while clarifying the processes driving interdisciplinary communications during the mHTIs.

## Introduction

The need for interdisciplinary thinking and communication has gained prominence in team science given the need for research teams to work together to solve complex scientific problems [1–3]. Interdisciplinary thinking indicates the capacity to integrate knowledge and research approaches from two or more disciplines to understand a phenomenon or solve a problem that could not have been achieved through a single discipline [4]. Given the importance of interdisciplinarity in tackling complex scientific challenges, such as those related to translational science, various initiatives have emerged to foster interdisciplinary thinking by intentionally bringing researchers from different disciplines together to collaborate [5–11]. Much evidence-based guidance has emerged regarding how to successfully facilitate interdisciplinary thinking and collaboration for such initiatives [3, 12–14]

In this study, we evaluated the mHealth Training Institutes (mHTI), one such program designed to develop scientists capable of engaging in and spearheading interdisciplinary efforts to develop effective mobile health (mHealth) solutions. The importance of interdisciplinary thinking within the area of mHealth has been highlighted by studies which have found that mHealth solutions developed from a consideration of broader perspectives are more effective than those developed with just a single perspective [15–18]. Thus, one goal of this study is to understand whether the mHTI program can foster interdisciplinary conversations and be a model for understanding how interdisciplinary thinking and collaborations can be nurtured. Another goal is to apply and evaluate how novel longitudinal, model-based social network analysis techniques can evaluate this and other similar programs. Social network analyses are useful for studying team activities because they help identify influential members (such as decision makers and thought leaders) based on centrality measures and clarify factors underlying temporal changes in collaboration patterns.

### Prior Evaluations of Programs Fostering Team Science Skills

Most evaluations of interdisciplinary training initiatives – such as the one conducted by Read et al. [19] – are predominantly cross-sectional and utilize only pre- and post-program assessments while neglecting more granular longitudinal shifts in team processes [20]. Evolving social networks deserve special consideration and analysis in the evaluation of team science since they represent the structure of the communication channels through which collaborative innovation and creativity occur [21–23]. Yet, evaluations involving analyses of network structures are uncommon. Thus, we used a model-based social network analysis to identify whether and how interdisciplinary collaborations occur and what factors contribute to their formation and persistence. Our models gauged the extent to which participants in the program (scholars) interacted with those who are similar (homophily) or different in certain attributes (heterophily).

To our knowledge, few network-based evaluations of team science skills or interdisciplinary initiatives have utilized such a model-based approach; evaluations that do use social network analyses, like those by Wu and Duan [24], Roelofs et al. [20], and Patterson et al. [25], have been exploratory and qualitative, applying network visualizations and descriptive statistics instead of inferential network models. Okamoto et al. [22] conducted a cross-sectional, model-based social network analysis of scientific collaboration but acknowledged the need for longitudinal analysis to better understand how participant characteristics influence change in collaboration patterns between prior and current networks. Thus, we use a longitudinal, model-based approach that allows exploration of how interdisciplinary team member interactions/collaborations change over time during project development. By doing so, we aim to uncover insights about factors that play a role in the formation and persistence of interpersonal team collaborations.

One of the goals of the mHTI is to ensure conversations among scholars of different disciplines that could translate into productive future transdisciplinary collaborations; these conversations are vital for the formation of interpersonal relationships that will facilitate interdisciplinary scientific progress [26]. Therefore, we hypothesize that if the mHTI met this goal and successfully supported interdisciplinary conversations, the formation and persistence of work (project-based) conversations should not be driven by disciplinary homophily. In other words, throughout the mHTI, participants should be just as likely to engage in project-based conversations with others from different disciplines as they are with those from their own discipline. This effect should exist even after accounting for participants’ preexisting baseline levels of openness to and institutional support for interdisciplinary work. We also hypothesize that a successful mHTI will not display disciplinary homophily for fun (non-project-based) conversations among the participants since, ideally, these conversations could pave the way toward more interdisciplinary conversations and collaborations.

## Materials and Methods

### The mHealth Training Institutes

The NIH-funded mHTI [27] is an immersive training program intended to foster the development of scientists who can engage in and lead interdisciplinary collaborations that develop mHealth solutions to complex healthcare problems. We describe the application and selection process in the Supplementary Materials (S7). The key objectives of the mHTI are to increase the selected scholars’ appreciation of different disciplinary perspectives and methodologies through the interdisciplinary networking and conversations they have with one another (which is the focus of our study), develop their self-efficacy to execute transdisciplinary mHealth collaborations, and heighten their engagement in developing transdisciplinary mHealth solutions. Although we focus on the first goal, the goals are all related given that interpersonal relationships within team science can increase scientific productivity outcomes [26]. The annual, weeklong program connects behavioral scientists, nurses and physicians, computer and data scientists, and engineers in a deliberate manner, using a cohesive pedagogical framework to promote a shared vocabulary, transdisciplinary orientation, and grounding in cutting-edge research methods and analytic approaches. The shared experience of working on a team science research project is designed to offer an experiential way to cultivate openness, mutual trust, and respect for differing disciplinary expertise and perspectives.

The mHTI’s blended-learning approach uses a combination of didactic deep dives led by mHTI faculty (for all scholars, senior or junior) and mentor-facilitated team project work. Grouped into five multidisciplinary teams each led by two experienced faculty mentors, the scholars identify a health problem and develop a proposal for an mHealth solution during the institute. The team projects culminate in a final oral capstone presentation that is subsequently converted into a brief research proposal scored by independent scientists using NIH review criteria.

### Network Measures

We studied the evolution of communication among interdisciplinary scholars embedded for 1 week at the mHTI during the years 2017−2019. Scholars had both structured time to discuss their projects and unstructured time built into common meals and evening social events to engage with one another. Assessments administered at the end of days 1, 3, and 5 of the mHTI surveyed individual scholars about their recent conversations with other scholars (response rates ranged from 89% to 100%). In both 2019 and 2018, a total of 29 scholars participated in the institute workshops, and in 2017 a total of 35 scholars participated.

In the 2019 and 2018 mHTI, scholars were asked to identify those with whom they had (1) project-related discussions and (2) an enjoyable/fun conversation about any topic (“fun conversations”). In the 2017 mHTI, only project-related conversations were recorded. Scholars were shown the names of all other scholars and faculty mentors attending the mHTI that year and were asked to indicate those with whom they spoke. The survey asked scholars about conversations they had in the period from the previous survey time point to the end of the day when they took the survey.

We constructed two networks depicting both project-related and fun communication among the scholars (excluding mentors) for 2019 and 2018 and one network depicting only the project-related discussions for 2017. We created an *N* by *N* adjacency network matrix, where *N* is the number of scholars, for each conversation type per day that depicts the conversations from one scholar to another. If scholar *A nominated* scholar *B as* a conversation partner of that day, the *A*th row *and B*th column entry of the matrix is 1 and 0 otherwise. Each row of the matrix corresponds to a particular scholar *A*, while each column corresponds to a particular scholar B. The networks are directed networks because while scholar *A* may have nominated scholar *B*, scholar *B* may not have nominated scholar *A* (i.e., the adjacency matrix is not necessarily symmetric). Scholars would only nominate conversations they believed that mattered (hence the directed ties) and classify them into categories they perceived appropriate. All scholars are actors in the project- and fun-based network conversation types and have their own attributes and network properties. To show how the two types of networks evolved over time, we present descriptive statistics and visualizations of the networks in the following section. These descriptive statistics have substantive meaning. For example, degree centrality identifies those with the most links to other scholars in the network and is a measure of the scholar’s prominence or structural importance in a network. A high degree of centrality could indicate power, influence, control, or status as reflected by the number of indegree (inbound) and outdegree (outbound) links. Another measure, closeness centrality, calculates the shortest paths between all nodes and assigns each node a score based on its sum of shortest paths. Nodes with high closeness centrality have the potential to be good “broadcasters” or influencers in a single cluster.

### Participant Attributes

#### Team

Scholars in each year were assigned to five different project teams whose members remained constant throughout the training. Teams were intentionally constituted to include all disciplines represented at the institute. Creation of this team variable enabled us to examine whether institute scholars networked with others outside of their team.

#### Career Stage (STG)

Scholars reported their career stage, and this variable was dichotomized such that graduate students and postdoctoral fellows were classified as early career scholars and assistant, associate or full professors were classified as late career scholars. Creation of this variable allowed us to evaluate whether the training institute fostered relationships between senior scientists and more junior investigators, consistent with recommendations for team science collaborations [22].

#### Gender (GEN)

Gender was recorded as to whether the scholar self-identified as male or female or declined to state.

#### Discipline (DSC)

Scholars reported their primary scholarly discipline. Disciplines were categorized as: Computer Science/Engineering/Data Science (CS), Medicine/Nursing (MED), Psychology (PSY), and Public Health/Others (OTH). The Psychology category included subdisciplines of psychology such as clinical or social psychology. The OTH category included fields such as epidemiology, management science, health economics, and human development and family studies.

#### Openness to Interdisciplinary Collaboration (“Openness”)

Scholars completed a six-item scale that reflected their openness to interdisciplinary collaboration. This scale was adapted from the behavior change collaboration activities index [28]. Scholars provided responses ranging from “completely false” to “completely true” to items with a 7-point Likert scale such as “I have a readiness to collaborate with researchers outside my field.” The mean scores for each scholar were calculated and used as covariates in the models since this variable could influence the likelihood of transdisciplinary conversations during the mHTI. The internal consistency of the measures was reasonable with Cronbach’s alpha of 0.62, 0.75, and 0.73 in 2017, 2018, and 2019, respectively. The full set of items and additional details on the psychometric properties of the items are provided in the Supplementary Materials (S3).

#### Perceived Institutional Support for mHealth-Specific Interdisciplinary Collaboration (“Support”)

Scholars also completed an 18-item scale developed for this project with 5-point Likert-type items which reported levels of perceived institutional support for mHealth-specific interdisciplinary collaboration. Scholars provided responses ranging from “punished” to “rewarded” to items such as “Collaboration on mHealth projects with researchers outside my institution who come from disciplines or fields of study different from my own.” The mean scores for each scholar were also calculated and used as covariates. Internal consistency of the Support measure was satisfactory, with Cronbach’s alpha of 0.91, 0.90, and 0.93 in 2017, 2018, and 2019, respectively. Additional details on the psychometric properties of the items are provided in the Supplementary Materials (S4).

### Analytic Method: Separable Temporal Exponential Random Graph Models

To investigate what factors contributed to the formation and persistence of communications among the participating scholars during the mHTI, we used separable temporal exponential random graph models (STERGMs) [29]. STERGMs are an extension of exponential random graph models (ERGMs) that explain the structure of networks using functions of the observed network and nodal and edge attributes. Whereas ERGMs can characterize network structures at a single point in time, STERGMs allow us to go beyond examining single occasions of one or more independent networks and to instead investigate dynamic team processes in the same networks examined repeatedly over time. STERGMs have been applied to examine changes in organizational collaboration [30] and fluctuations in team performance on simulated long-duration space exploration [31]. Since our network panel data include three observations of the same network, we used these models to understand evolving communication patterns among scholars within the mHTI.

A key advantage of STERGMs is they allow for specification of two separate models – one for tie formation and one for tie persistence (or dissolution). This flexibility is particularly useful when the processes underlying the formation of ties may be markedly different from those of the underlying dissolution of ties. The outcomes of the formation and persistence models are the log-odds of a given tie existing at time *t+1* if it did not exist at time *t* and the log-odds of a given tie existing at time *t+1* if it did exist at time *t*, respectively.

For each conversation type (project-related vs fun), we specified three different models for each of the 3 years of training institute data. These models were estimated using the *tergm* package in R [32], and each model builds on the previous one. No models were fit for fun conversations in 2017, since only project-related conversations were recorded for that year’s mHTI. Five 2017 scholars were excluded from the analysis due to missing data for one or more variables. The threshold for statistical significance was set at 0.05. The Supplementary Materials (S6) contains the link where the script can be found.

#### Model 1: Baseline Model

**Fig. 1.**
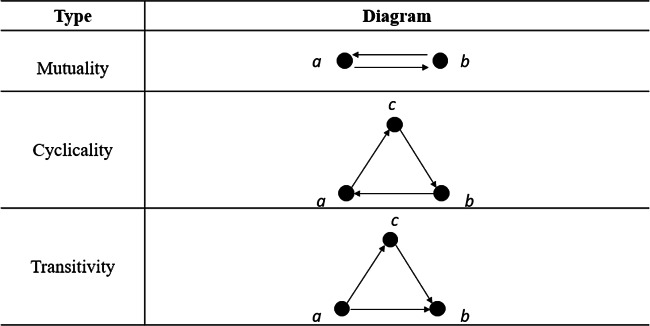
Visualizations of mutuality, cyclicality, and transitivity. These types of network relationships are shown for individual actors *a* and *b* in which an arrow denotes a directed tie.

We specified a baseline model to examine the basic structure of the communication network and its formation and persistence structure when no other background variables are considered. The baseline model contains three variables that represent network characteristics: (1) mutuality: counts of mutual dyads, (2) cyclicalities: counts of nonhierarchical triangles, and (3) transitivities: counts of hierarchical triangles. Cyclicalities counts the number of conversations from scholar *a* to scholar *b* such that there is also a conversation from scholar *b* to some scholar *c*, and from scholar *c* to scholar *a*. Transitivities counts the number of conversations from scholar *a* to scholar *b* such that there is also a conversation from scholar *a* to some scholar *c*, and from scholar *c* to scholar *b*. The transitivities term substantiates a hierarchical relationship in which scholar *a* talks to scholar *c* who talks to scholar *b* (suggesting that *a* defers to *c* who defers to *b*, whom both scholars want to talk to) whereas the cyclicalities term shows a more egalitarian, cyclical relationship (i.e., the three scholars talk to one another). The use of directed ties in our network allows us to capture these network substructures. Fig. 1 depicts these three different variables. The regression coefficients of these variables help us understand the structures of the formation and persistence of the communication network.

#### Model 2: Team, Stage, Discipline, and Gender Homophily Effects

Model 2 includes all terms from Model 1 plus a homophily effect by team membership. This homophily effect enables us to understand whether social network ties, that is, conversations between two scholars, are more likely to form or persist between two scholars from the same team. Model 2 also includes homophily effects by career stage, discipline, and gender. These additional terms allow us to understand whether ties are more likely to form and/or persist between scholars who are in the same career stage, discipline, or of the same gender. The former two effects are particularly informative since one goal of the mHTI is to ensure conversations among scholars from different disciplines and stages of their careers. Looking at these terms can help us evaluate the extent to which the institute initially succeeded in this goal, while controlling for the team homophily effect; based on our hypothesis, we would expect these discipline and career homophily terms to not be statistically significant if the mHTI met the goal. The team homophily effect is important to control for since team homophily effects were likely to be present due to the design of the program (i.e., that members of a given team were expected to work on their shared project throughout the week). Additionally, it would be informative to understand whether gender homophily effects were present in the interactions during the program.

#### Model 3: Openness to Transdisciplinary Collaboration and Institutional Support Outdegree

Model 3 includes Openness and Support as additional covariates in the formation and persistence models. We examined the effects of these variables to see whether scholars were a self-selected group who already possessed high levels of institutional support or willingness to collaborate with others from different backgrounds. If such self-selection was present, the presence of interdisciplinary conversation that we identified could be a byproduct of these pre-institute characteristics rather than a result of the training institute’s program and design. Hence, examining and controlling for these effects helps us validate the impact of the mHTI more accurately.

## Results

### Scholars’ Background Characteristics

Table 1 presents the scholars' background characteristics. The number of scholars per team ranged from 5 to 7. There were generally more late- than early-stage career scholars and more female than male scholars. For instance, in 2019, about 59% were late-stage career scholars and about 62% were female. Disciplinary representation was even over 3 years, except for overrepresentation of CS in 2017 (37.1%) and MED in 2018 (37.9%). The Openness and Support scores had similar distributions over the years, respectively.

**Table 1.** Scholars’ background characteristics and centrality measures in networks

		2017	2018	2019
Background		Freq. (%)	Freq. (%)	Freq. (%)
Team	1	5 (16.7)	6 (20.7)	6 (20.7)
	2	7 (23.3)	6 (20.7)	6 (20.7)
	3	6 (20.0)	6 (20.7)	5 (17.2)
	4	7 (23.3)	5 (17.2)	7 (24.1)
	5	5 (16.7)	6 (20.7)	5 (17.2)
Stage	Early	8 (26.7)	9 (31.0)	12 (41.4)
	Late	22 (73.3)	20 (69.0)	17 (59.0)
Gender	Female	17 (56.7)	13 (44.8)	18 (62.1)
	Male	13 (43.3)	16 (55.2)	11 (38.0)
Discipline^1^	CS	10 (33.3)	5 (17.2)	8 (27.6)
	MED	10 (33.3)	11 (37.9)	8 (27.6)
	PSY	5 (16.7)	8 (27.6)	7 (24.1)
	OTH	5 (16.7)	5 (17.2)	6 (20.7)

		Mean (SD)	Mean (SD)	Mean (SD)
Openness^2^		5.37 (0.82)	5.14 (1.08)	5.31 (1.05)
Support^3^		4.10 (0.55)	3.73 (0.49)	3.83 (0.49)

Centrality		Day 1	Day 3	Day 5	Correlation	
		Mean (SD)	Mean (SD)	Mean (SD)	1 & 3	3 & 5
Type 1: project-based conversation						
2017	Outdegree	3.97 (2.61)	5.30 (4.81)	4.97 (4.59)	0.59	0.72
	Indegree	3.97 (1.16)	5.30 (1.64)	4.97 (1.56)	0.60	0.33
	Closeness	0.25 (0.18)	0.38 (0.21)	0.35 (0.21)	0.48	0.65
	Betweenness	32.63 (40.48)	27.83 (31.95)	32.60 (44.79)	0.57	0.56
2018	Outdegree	4.10 (2.92)	6.14 (3.81)	6.14 (4.53)	0.73	0.70
	Indegree	4.10 (1.32)	6.14 (1.77)	6.14 (1.77)	0.35	0.70
	Closeness	0.00 (0.00)	0.43 (0.19)	0.43 (0.21)	–	0.44
	Betweenness	22.24 (38.98)	25.24 (32.63)	23.17 (28.15)	0.46	0.79
2019	Outdegree	5.55 (3.52)	5.38 (2.43)	5.93 (4.28)	0.86	0.53
	Indegree	5.55 (1.15)	5.38 (1.35)	5.93 (1.62)	0.44	0.62
	Closeness	0.41 (0.14)	0.34 (0.17)	0.39 (0.19)	0.61	0.27
	Betweenness	35.65 (51.02)	35.76 (46.11)	33.21 (43.18)	0.47	0.31
Type 2: project-based conversation						
2018	Outdegree	5.38 (4.83)	8.76 (6.25)	9.14 (6.93)	0.60	0.80
	Indegree	5.38 (2.37)	8.76 (3.63)	9.14 (2.72)	0.57	0.78
	Closeness	0.38 (0.25)	0.50 (0.25)	0.51 (0.26)	0.69	0.64
	Betweenness	19.31 (23.20)	16.00 (18.25)	15.24 (14.62)	0.48	0.72
2019	Outdegree	9.31 (4.22)	10.66 (5.66)	12.00 (7.61)	0.52	0.56
	Indegree	9.31 (3.53)	10.66 (2.47)	12.00 (3.46)	0.77	0.60
	Closeness	0.59 (0.13)	0.59 (0.18)	0.58 (0.26)	0.68	0.62
	Betweenness	17.93 (17.64)	16.14 (12.81)	12.55 (11.12)	0.58	0.45

Outdegree and indegree indicate the number of ties that are sent and received by the scholars, which shows each scholar’s activity and popularity in a given network, respectively. Closeness measures how close each node is to other nodes in the network, defined as the reciprocal of farness where the farness is the average distance from a node to all other nodes. Betweenness measures the number of times that a node, that is, scholar, lies on the shortest path between other scholars.

1 CS, Computer Science/Engineering/Data Science; MED, Medicine/Nursing; PSY, Psychology; OTH, Public health/Others.

2 Openness scale: Cronbach’s α = 0.62 in 2017, 0.75 in 2018, and 0.73 in 2019.

3 Support scale: Cronbach’s α = 0.91 in 2017, 0.90 in 2018, and 0.93 in 2019.

### Network Characteristics

Table 1 also lists the four centrality measures for each network. For the project-based communication network, the number of indegree and outdegree ties tended to increase from day 1 to day 5, except for 2017 when the number of ties somewhat decreased from day 3 to day 5. The fun-based communication network grew with an increasing average number of outdegree and indegree ties from day 1 to day 3 and from day 3 to day 5. As can be seen in the last two columns indicating the correlations of centrality measures between adjacent days’ networks, central scholars in previous days’ networks maintained their central positions in the later days’ networks, and this pattern was consistently shown in all years.

**Fig. 2.**
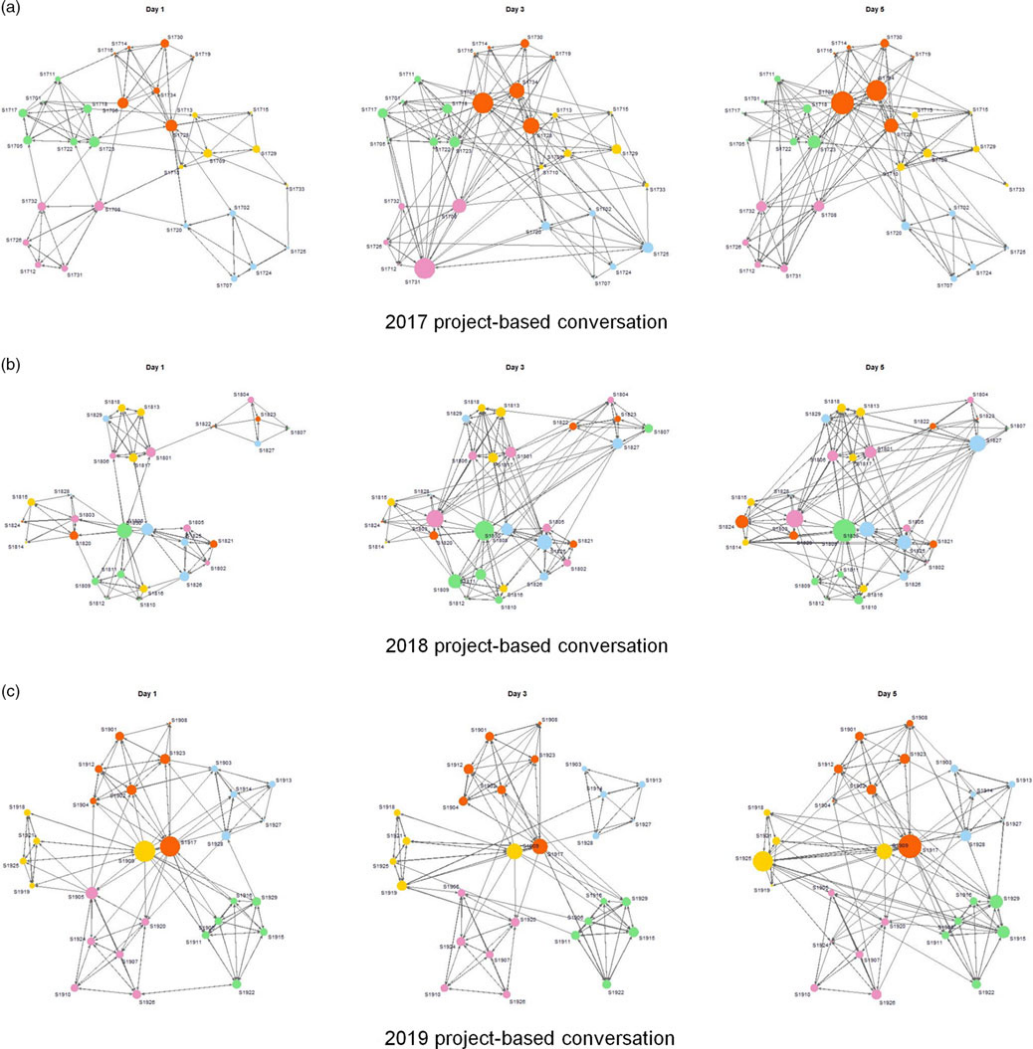
Network visualizations of team homophily for project-based conversations. Circles indicate scholars, sizes of circles represent the level of scholar’s activeness (outdegree) in the network, and arrows represent conversation ties. Colors in circles indicate team membership; pink for team 1, green for team 2, yellow for team 3, red for team 4, sky blue for team 5.

Table 2 lists additional network measures (edges, mutual, cyclicalities, transitive, and homophily) that describe each communication network’s structures. We observed some general patterns over 3 years: (1) scholars tended to have conversations with more scholars as the training institute went on; (2) scholars were more active in fun conversations (i.e., more edges) than in project-based conversations; (3) the number of transitive triangles was larger than the number of cyclical triangles in both conversation types, indicating that the scholars tended to have conversations in hierarchical clusters rather than in egalitarian clusters (i.e., in triads, scholars tended to converse with one particular person instead of conversing with one another more equally); (4) all of the networks suggested homophily in stage and gender since based on the values in parentheses, almost or more than 50% of conversations occurred within those same attributes. For team homophily, however, the proportions of the within-team ties were larger in project-based conversations than in have-fun conversations. This makes sense given the team project-based nature of the mHTI and means that team members’ discussions of their project did not crowd out the broader group networking process that was also an mHTI goal. Compared with the 2017 and 2019 project-based conversation networks, the 2018 project-based conversation network had a far smaller number of within-team ties. Fig. 2 displays the team homophily of the project-based networks. All other network visualizations with different homophily types are presented in the Supplementary Materials (S1).

**Table 2.** Network structure measures of the scholars’ networks

	Type 1: project-based conversation					
2017	Day 1	Day 3	Day 5			
Str	Val (%)	Val (%)	Val (%)	Type 2: have-fun conversation		
Edges	119 (13.7)	159 (18.3)	149 (17.1)			
Mutual	31	33	37			
Cyclicalities	67	92	86			
Transitive	221	313	327			
Same team	97 (81.5)	92 (57.9)	95 (63.8)			
Same STG	67 (56.3)	88 (55.4)	85 (57.1)			
Same GEN	60 (50.4)	82 (51.6)	69 (46.3)			
Same DSC	24 (20.2)	41 (25.8)	39 (26.2)			

2018	Day 1	Day 3	Day 5	Day 1	Day 3	Day 5
Str	Val (%)	Val (%)	Val (%)	Val (%)	Val (%)	Val (%)
Edges	119 (14.7)	178 (21.9)	178 (21.9)	156 (19.2)	254 (31.3)	265 (32.6)
Mutual	39	65	53	37	72	69
Cyclicalities	81	145	130	101	225	218
Transitive	293	570	530	341	1220	1279
Same team	31 (26.1)	46 (25.8)	43 (24.2)	26 (16.7)	55 (21.7)	53 (20.0)
Same STG	65 (54.6)	100 (56.2)	100 (56.2)	98 (62.8)	147 (57.9)	159 (60.0)
Same GEN	58 (48.7)	77 (43.3)	83 (46.6)	67 (43.0)	120 (47.2)	117 (44.2)
Same DSC	22 (18.5)	40 (22.5)	40 (22.5)	49 (31.4)	68 (26.8)	69 (26.0)

2019	Day 1	Day 3	Day 5	Day 1	Day 3	Day 5
Str	Val (%)	Val (%)	Val (%)	Val (%)	Val (%)	Val (%)
Edges	161 (19.8)	156 (19.2)	172 (21.1)	288 (35.4)	309 (38.0)	348 (42.8)
Mutual	58	64	56	103	97	106
Cyclicalities	135	141	133	283	292	299
Transitive	495	510	537	1347	1703	2582
Same team	125 (77.6)	130 (83.3)	119 (69.2)	95 (33.0)	100 (32.4)	97 (27.9)
Same STG	77 (47.8)	76 (48.7)	82 (47.7)	139 (48.3)	154 (49.8)	163 (46.8)
Same GEN	75 (46.6)	83 (53.2)	93 (54.1)	164 (57.0)	176 (57.0)	188 (54.0)
Same DSC	28 (17.4)	30 (19.2)	35 (20.4)	68 (23.6)	77 (24.9)	84 (24.1)

DSC, discipline; GEN, gender; STERGM, separable temporal exponential random graph model; STG, stage.

The percentage in the "Edges" rows (i.e., the number in parentheses) represents the number of edges in the network divided by the number of possible edges in the network, which is referred to as "density" in the social network analysis literature. The percentage in the "Same-" rows indicates the number of edges from scholars with the same attribute divided by the number of edges in the network. This can be considered a measure of "homophily."

### Model Analysis Results

The convergence of Model 3 for all years was assessed using trace plots and the distributions of the parameter estimates. The trace plots (presented in Supplementary Materials S2) suggest that the estimates of the parameters are stable and that convergence was reached.

The goodness-of-fit of these models was also assessed by simulating large numbers of networks from the estimated models and plotting the distributions of the sufficient statistics of those simulated networks against the sufficient statistics from the observed network. In a good fit, the observed sufficient statistics should be close to the median of the sample sufficient statistics [33]. These boxplots, displayed in Supplementary Materials S2, suggest the satisfactory goodness-of-fit of our models.

In general, an antiegalitarian, hierarchical dynamic influenced the formation and persistence of both kinds of conversations. This is shown from the cyclicalties and transitiveties terms in the models, which are negative and positive, respectively, across the models for all the years. These hierarchical relationships were more likely than egalitarian relationships to form and persist in the mHTI.

The results for all years are presented in Tables 3–5. Generally, the parameter estimates are consistent across different models.

**Table 3.** STERGM result: 2017 project-based conversation

	Model 1		Model 2		Model 3	
	Est	(SE)	Est	(SE)	Est	(SE)
Formation						
Edges	*−3.59*	(0.25)**	*−3.57*	(0.30)***	*−5.63*	(0.99)***
Mutual	*1.52*	(0.28)***	*0.99*	(0.33)**	*0.96*	(0.33)**
Cyclicalities	*−0.33*	(0.11)**	*−0.35*	(0.11)***	*−0.31*	(0.11)**
Transitiveties	*1.04*	(0.21)***	*1.00*	(0.21^)***^	*0.90*	(0.22)***
Team homophily			*1.03*	(0.30)***	*1.06*	(0.30)***
STG homophily			*−*0.20	(0.20)	*−*0.19	(0.20)
GEN homophily			0.01	(0.20)	*−*0.02	(0.20)
DSC homophily			*0.54*	(0.21)*	*0.55*	(0.21)**
Openness					*0.30*	(0.13)*
Support					0.11	(0.17)
Persistence						
Edges	*−0.52*	(0.26)*	*−0.81*	(0.41)*	*−9.71*	(1.92)***
Mutual	0.62	(0.49)	0.07	(0.56)	0.40	(0.61)
Cyclicalities	*−0.59*	(0.19)**	*−0.69*	(0.21)***	*−0.67*	(0.21)**
Transitiveties	*1.44*	(0.22)***	*1.09*	(0.24)***	*1.05*	(0.26)***
Team homophily			*1.92*	(0.40)***	*2.69*	(0.47)***
STG homophily			0.15	(0.32)	0.11	(0.36)
GEN homophily			*−*0.48	(0.32)	*−*0.41	(0.36)
DSC homophily			0.21	(0.36)	0.37	(0.40)
Openness					*1.15*	(0.21)***
Support					0.53	(0.33)

DSC, discipline; GEN, gender; STERGM, separable temporal exponential random graph model; STG, stage.

*** *p* < 0.001; **p < 0.01; *p < 0.05. Statistically significant terms are italicized.

**Table 4.** STERGM result: 2018 conversations

	Project-based conversations						Have-fun conversations					
	Model 1		Model 2		Model 3		Model 1		Model 2		Model 3	
	Est	(SE)	Est	(SE)	Est	(SE)	Est	(SE)	Est	(SE)	Est	(SE)
Formation												
Edges	*−4.11*	(0.30)***	*−4.14*	(0.35)***	*−4.10*	(0.83)***	−1.56	(1.01)	−1.68	(1.07)	*−2.88*	(1.17)*
Mutual	*3.16*	(0.34)***	*3.12*	(0.33)***	*3.17*	(0.33)***	*1.64*	(0.19)***	*1.60*	(0.20)***	*1.61*	(0.20)***
Cyclicalities	*−0.81*	(0.16)***	*−0.81*	(0.16)***	*−0.78*	(0.16)***	*−0.99*	(0.13)***	*−0.95*	(0.17)***	*−1.01*	(0.14)***
Transitiveties	*1.64*	(0.28)***	*1.64*	(0.29)***	*1.58*	(0.28)***	0.70	(1.00)	0.68	(1.04)	0.67	(0.93)
Team homophily			0.02	(0.26)	0.10	(0.26)			0.21	(0.25)	0.23	(0.25)
STG homophily			0.16	(0.19)	0.13	(0.19)			−0.04	(0.14)	−0.02	(0.14)
GEN homophily			−0.20	(0.19)	−0.20	(0.20)			0.23	(0.14)	0.24	(0.14)
DSC homophily			0.14	(0.21)	0.15	(0.21)			0.08	(0.17)	0.11	(0.17)
Openness					*0.21*	(0.09)*					−0.09	(0.08)
Support					−0.31	(0.20)					*0.45*	(0.16)**
Persistence												
Edges	0.26	(0.39)	0.12	(0.49)	0.10	(1.62)	−0.06	(0.28)	−0.10	(0.32)	−0.22	(0.86)
Mutual	*1.33*	(0.47)**	*1.10*	(0.49)*	*1.15*	(0.47)*	*0.88*	(0.24)***	*0.62*	(0.25)*	*0.62*	(0.25)*
Cyclicalities	*−0.92*	(0.24)***	*−0.83*	(0.24)***	*−0.85*	(0.24)***	*−0.63*	(0.11)***	*−0.59*	(0.11)***	*−0.59*	(0.11)***
Transitiveties	*1.40*	(0.32)***	*1.36*	(0.33)***	*1.35*	(0.33)***	*1.09*	(0.24)***	*0.91*	(0.24)***	*0.90*	(0.25)***
Team homophily			*1.08*	(0.49)*	*1.06*	(0.47)*			*1.15*	(0.23)***	*1.14*	(0.24)***
STG homophily			−0.22	(0.32)	−0.23	(0.32)			−0.09	(0.18)	−0.09	(0.18)
GEN homophily			0.31	(0.33)	0.30	(0.32)			0.00	(0.18)	−0.00	(0.19)
DSC homophily			0.05	(0.38)	0.04	(0.37)			0.37	(0.22)	0.36	(0.22)
Openness					0.05	(0.15)					−0.03	(0.08)
Support					−0.06	(0.41)					0.07	(0.20)

DSC, discipline; GEN, gender; STERGM, separable temporal exponential random graph model; STG, stage.

*** *p* < 0.001; **p < 0.01; *p < 0.05. Statistically significant terms are italicized.

**Table 5.** STERGM result: 2019 conversations

	Project-based conversations						Have-fun conversations					
	Model 1		Model 2		Model 3		Model 1		Model 2		Model 3	
	Est	(SE)	Est	(SE)	Est	(SE)	Est	(SE)	Est	(SE)	Est	(SE)
Formation												
Edges	*−3.76*	(0.29)***	*−4.27*	(0.40)***	*−11.36*	(1.64)***	−1.57	(1.04)	−1.62	(1.06)	*−2.89*	(1.16)*
Mutual	*2.18*	(0.36)***	*1.40*	(0.43)**	*1.33*	(0.44)**	*1.64*	(0.19)***	*1.60*	(0.19)***	*1.61*	(0.19)***
Cyclicalities	*−0.37*	(0.17)*	*−0.34*	(0.17)*	*−0.44*	(0.17)**	*−0.98*	(0.14)***	*−0.95*	(0.16)***	*−1.00*	(0.13)***
Transitiveties	*0.79*	(0.25)**	*0.76*	(0.26)**	*0.59*	(0.26)*	0.71	(1.02)	0.62	(1.04)	0.66	(0.94)
Team homophily			*2.15*	(0.48)***	*2.64*	(0.51)***			0.21	(0.25)	0.24	(0.25)
STG homophily			0.11	(0.26)	0.11	(0.26)			−0.04	(0.14)	−0.01	(0.14)
GEN homophily			*0.67*	(0.27)*	*0.90*	(0.28)**			0.23	(0.14)	0.25	(0.14)
DSC homophily			0.34	(0.29)	0.32	(0.29)			0.09	(0.17)	0.10	(0.17)
Openness					*0.59*	(0.15)***					−0.09	(0.08)
Support					*1.00*	(0.35) **					*0.45*	(0.16)**
Persistence												
Edges	−0.73	(0.46)	*−1.49*	(0.52)**	−2.02	(1.94)	−0.05	(0.29)	−0.10	(0.33)	−0.26	(0.87)
Mutual	*2.73*	(0.46)***	*1.43*	(0.62)*	*1.42*	(0.62)*	*0.88*	(0.24^)***^	*0.62*	(0.25)*	*0.62*	(0.25)*
Cyclicalities	−0.56	(0.29)	*−0.95*	(0.36)**	*−0.94*	(0.35)**	*−0.64*	(0.11)***	*−0.60*	(0.11)***	*−0.59*	(0.11)***
Transitiveties	*1.28*	(0.39)**	*0.93*	(0.37)*	*0.78*	(0.39)*	*1.10*	(0.25)***	*0.91*	(0.25)***	*0.90*	(0.24)***
Team homophily			*2.60*	(0.53)***	*2.87*	(0.59)***			*1.15*	(0.23)***	*1.15*	(0.23)***
STG homophily			0.03	(0.36)	0.03	(0.36)			−0.09	(0.18)	−0.09	(0.18)
GEN homophily			0.52	(0.36)	0.55	(0.37)			0.00	(0.19)	0.00	(0.18)
DSC homophily			0.61	(0.46)	0.64	(0.47)			0.36	(0.22)	0.36	(0.22)
Openness					0.29	(0.19)					−0.02	(0.08)
Support					−0.29	(0.44)					0.08	(0.20)

DSC, discipline; GEN, gender; STERGM, separable temporal exponential random graph model; STG, stage.

*** *p* < 0.001; **p < 0.01; *p < 0.05. Statistically significant terms are italicized.

#### Project-Based Conversations

In 2019, team homophily effects and gender homophily effects were positive and statistically significant in the formation model, suggesting that conversations between scholars from the same team or the same gender were more likely to form during the 2019 mHTI. However, only the team homophily effect was also positive and statistically significant in the persistence model. This finding suggests that conversations between scholars from the same team were likely to persist, whereas conversations between scholars from the same gender were just as likely to persist as those between scholars of different genders. The parameter estimates for Openness and Support were also positive in the formation model, suggesting that scholars with higher levels of openness or support were more likely to initiate conversations (although perhaps not to persist in those conversations) over the week. In contrast, in the 2018 mHTI there were no meaningful team or gender homophily effects present in the formation model. Unlike those in the 2019 mHTI, conversations between members of the same team or gender were not more likely to form than other kinds of conversations. Additionally, only the Openness effect was present and not the Support effect in the formation model. However, team-based conversations in 2018 were more likely to persist, like those in 2019.

The absence of stage and discipline homophily effects in 2018 and 2019 is also worth noting and is consistent with the mHTI goal of encouraging collaborations across career stage and discipline. Conversations between scholars of different stages or disciplines were just as likely to form or persist as conversations within those categories. In other words, after accounting for the team homophily effects, in the 2018 and 2019 mHTI, conversations between early-career and late-career scholars and between scholars of different disciplines were equally likely to form and persist.

However, in the 2017 mHTI, the discipline homophily effect was positive and statistically significant in the formation model, meaning that scholars were more likely to initiate conversation if they were from the same discipline. This effect was not present in the persistence model though, which means that same-discipline conversations were not any more likely to persist than different-discipline conversations. Like the results from the 2019 mHTI models, the results from the 2017 mHTI also include positive team homophily effects and Openness effects in both formation and persistence models.

#### Fun Conversations

There are also similarities in the formation and persistence models results for 2019 and 2018. No homophily effects were present in both years in the formation model. However, in the persistence model for both years, the team homophily effect was present, meaning that although team-based fun conversations were not any more likely to form, they were more likely to persist.

As noted in the results for the project-based conversations, the lack of stage and discipline homophily effects suggests that throughout the 2019 and 2018 mHTI, fun conversations within and across career stage and disciplinary lines were equally likely to form and persist.

## Discussion

We evaluated the social networks of the 2019, 2018, and 2017 mHTIs to uncover patterns and factors driving the formation and persistence of project-based and fun conversations, and particularly to characterize scholar interactions with others from different backgrounds. Using STERGMs, a novel longitudinal network analysis approach, we found that in 2019 and 2018, scholars did not speak exclusively to others from the same discipline or career stage. Rather, conversations across disciplinary and career stage boundaries were just as likely to form and persist as those within those boundaries. The only exception was the 2017 mHTI during which the discipline homophily effect was statistically significant. One possible explanation is that in response to 2017 scholar feedback, the mHTI organizers made changes to encourage more interdisciplinary conversations in subsequent years. Thus, while the 2017 mHTI did not meet the goal of ensuring interdisciplinary conversations, the 2018 and 2019 mHTI did. There were also some similarities across the years, such as the team homophily effects in 2017 and 2019 and the positive, statistically significant effects of the scholars’ attitudinal openness to interdisciplinary engagement on the formation and persistence of fun and project-based conversations.

### Implications

The results suggest that interdisciplinary communications were more likely to form in the 2018 and 2019 mHTIs, as compared to the 2017 mHTI. The growing trend toward communication across disciplines may reflect improvement in the pedagogical skills of the institute’s faculty or secular trends toward growing appreciation of interdisciplinarity in the mHealth community at large, or both: this study cannot establish the causal effects of the mHTI. Another limitation is that the perceived usefulness of these conversations is unknown. Regardless, it is important to note that interdisciplinary conversations were prominent during the mHTI, an impressive achievement given the considerable personal and disciplinary diversity of the mHTI participants. Unlike the shared understanding and seamless communication expected in traditional models of communication between like-minded scholars from the same discipline, coherence becomes more challenging in highly heterogeneous teams working on collaborative interdisciplinary research to tackle complex challenges. Despite the human tendency to cluster with like minds inherent in an intense, 1-week interdisciplinary bootcamp, the mHTI facilitated interdisciplinary communications. The selection process, team forming activities, and mentoring processes of the mHTI could be a promising model for other team science initiatives. Of note, it would be useful to use future mHTIs to investigate the gender homophily effect noted in the 2019 mHTI. Further, the evidence that junior and senior investigators were communicating (based on the lack of career stage homophily) is promising, given the benefits of collaborative relationships among newcomers (such as junior faculty) and incumbents (such as senior faculty) [22,34].

Unlike the team homophily effect, gender homophily was not an intent of the program’s design, although it is worth noting that the gender homophily effect was only present in the formation model (i.e., conversations between participants of the same gender were not any more likely to persist).

### Significance

This is the first network-based evaluation of the mHTI, an innovative training institute designed to foster interdisciplinary collaborations and team science skills. Our results suggest that the scholars within the mHTI were indeed having interdisciplinary conversations. The mHTI could be a model for practitioners and scholars to study and replicate in future efforts to foster interdisciplinary thinking and collaboration in the realm of team science. Our study also presents a novel methodological application of model-based social network analysis techniques to evaluate team science training initiatives. To our knowledge, most evaluations of such initiatives focus only on pre- and post-outcome measures without looking at the intermediate processes. The application of STERGMs helps us gain insights into how participants are interacting over time, beyond the information provided by simple network descriptive statistics. For example, while the descriptive statistics suggested some career stage homophily, the model-based results showed that this effect was not statistically significant in the formation or persistence of ties. We hope that this application will inspire other researchers and evaluators to also utilize more advanced social network techniques to assess team science dynamics. As noted by Roelofs et al. [20], social network analysis can be used not only in summative assessments as we have done here but also in ongoing formative assessments of the program to inform any corrections necessary to enhance collaboration. For example, if STERGMs reveal a lack of communication across disciplinary boundaries during the program, program leaders can adjust the program as necessary to foster more interdisciplinary conversations. Other evaluators can adapt these models to analyze their network data from similar trainings that can better inform the development and education of a translational workforce.

We suggest that future studies can include time-varying covariates. For example, throughout the institute, scholars reported their changing levels of their perceptions of the importance of mHealth initiatives, among other psychological constructs. In another paper we are preparing for publication, we use stochastic actor-oriented models [35] which can incorporate time-varying covariates to understand network and behavior dynamics. Such an analysis could also partially address a limitation of this study: that it cannot establish the causal effects of the mHTI. Future studies might also analyze the duration of these ties or conversations. Evaluating the duration of the conversations could allow us to better understand the extent to which scholars are truly communicating across disciplinary boundaries. By demonstrating the utility of a model-based social network analysis in evaluating a promising team science initiative, we hope that others will be inspired to undertake these studies in future evaluations of team science initiatives.

## Supplementary material

For supplementary material accompanying this paper visit http://dx.doi.org/10.1017/cts.2021.859. click here to view supplementary material
