# Supplementary material for: Fostering interdisciplinary collaboration: A longitudinal social network analysis of the NIH mHealth Training Institutes
Source: J Clin Transl Sci. 2021 Sep 20;5(1):e191. doi: 10.1017/cts.2021.859 (PMC8596066; doi:10.1017/cts.2021.859)
Supplement: Supplementary file 1 [file S2059866121008591sup001.docx]

**Supplementary Material**

*S1. Network graphs over three years of the mHTI*

Here we present graphical representations of the project-based and fun-based conversations during the 2017-2019 mHTI (Figures S1 to S5). In each network graph, scholars are indicated by circles with different sizes representing relative magnitudes of outdegree (i.e. reported more conversations with others), and solid arrows represent conversation ties that scholars had in the institute. To show group homophily over days, scholars are colored by their group membership based on scholar’s background characteristics as follows:

- Team membership: pink for team 1, green for team 2, yellow for team 3, red for team 4, sky blue for team 5.
- Stage: red for early career scholars and green for late career scholars.
- Gender: green for female scholars and red for male scholars.
- Discipline: pink for Computer Science/Engineering/Data Science, green for Medicine/Nursing, yellow for Psychology, and red for Public Health/Others.

*Figure S1. 2017 project-based conversations per day*

| **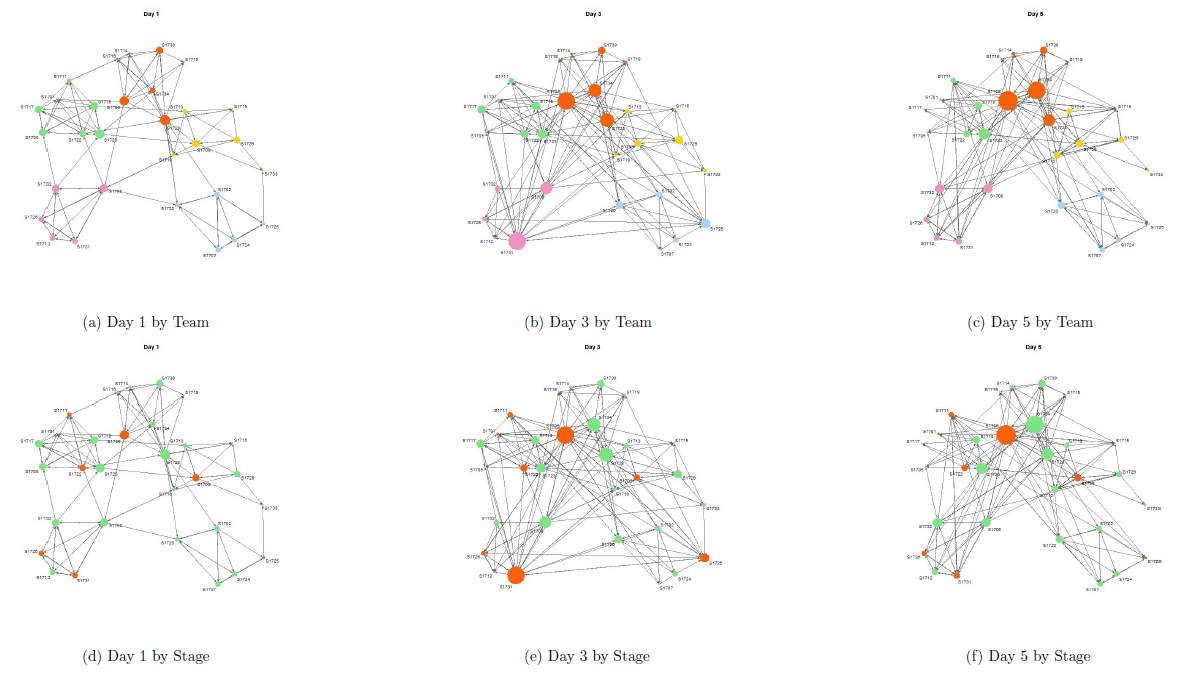** |
| --- |
| 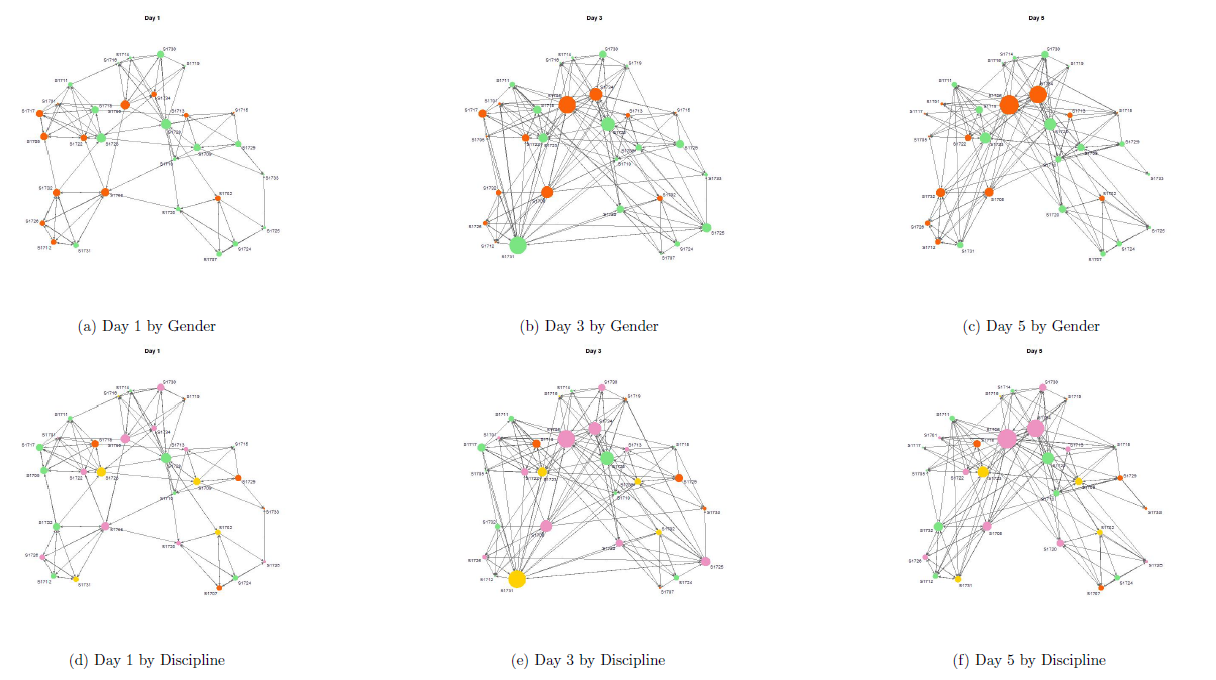 |

*Figure S2. 2018 project-based conversations per day*

| **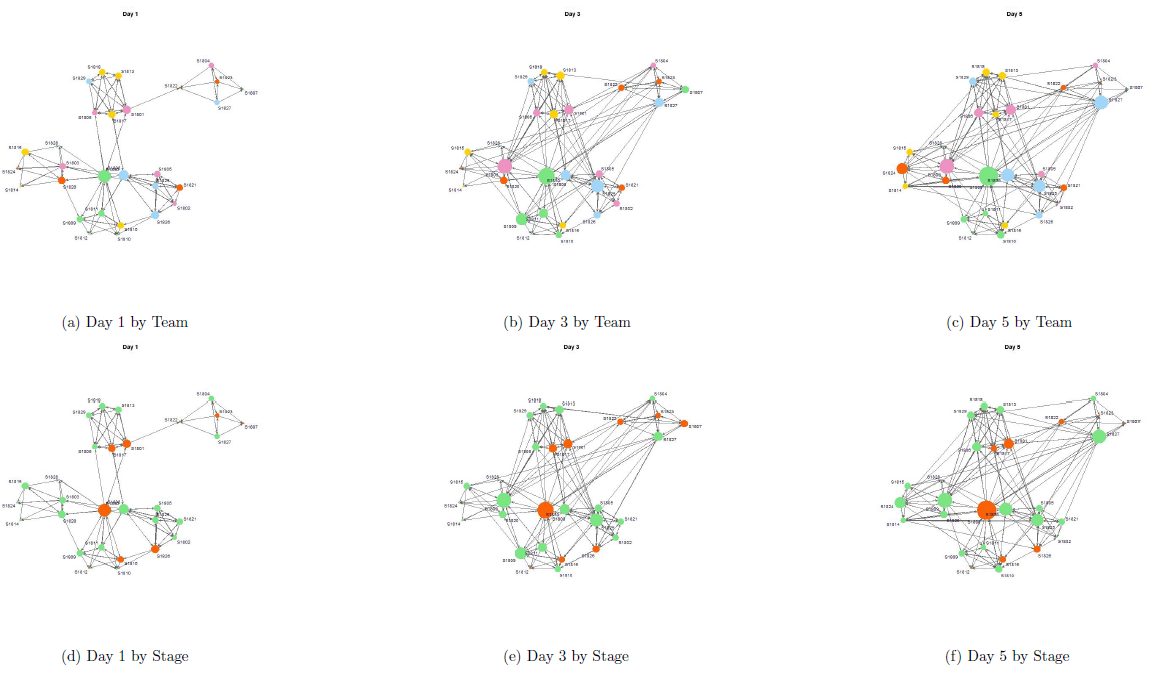** |
| --- |
| 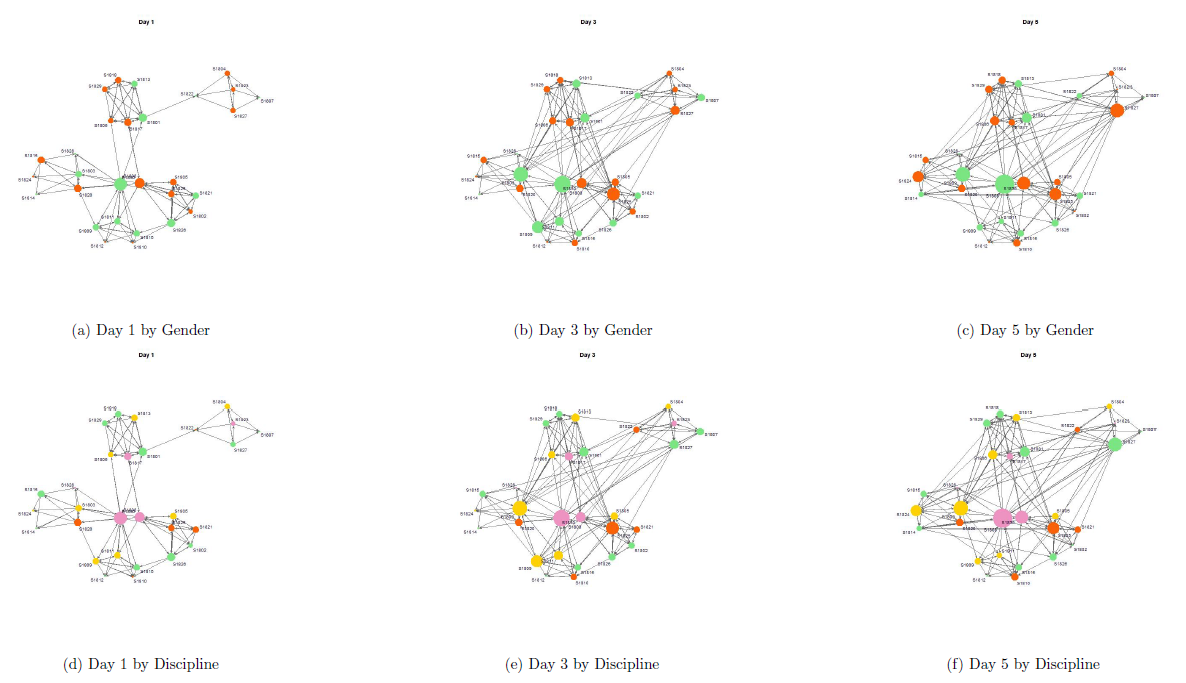 |

*Figure S3. 2018 fun conversations per day*

| **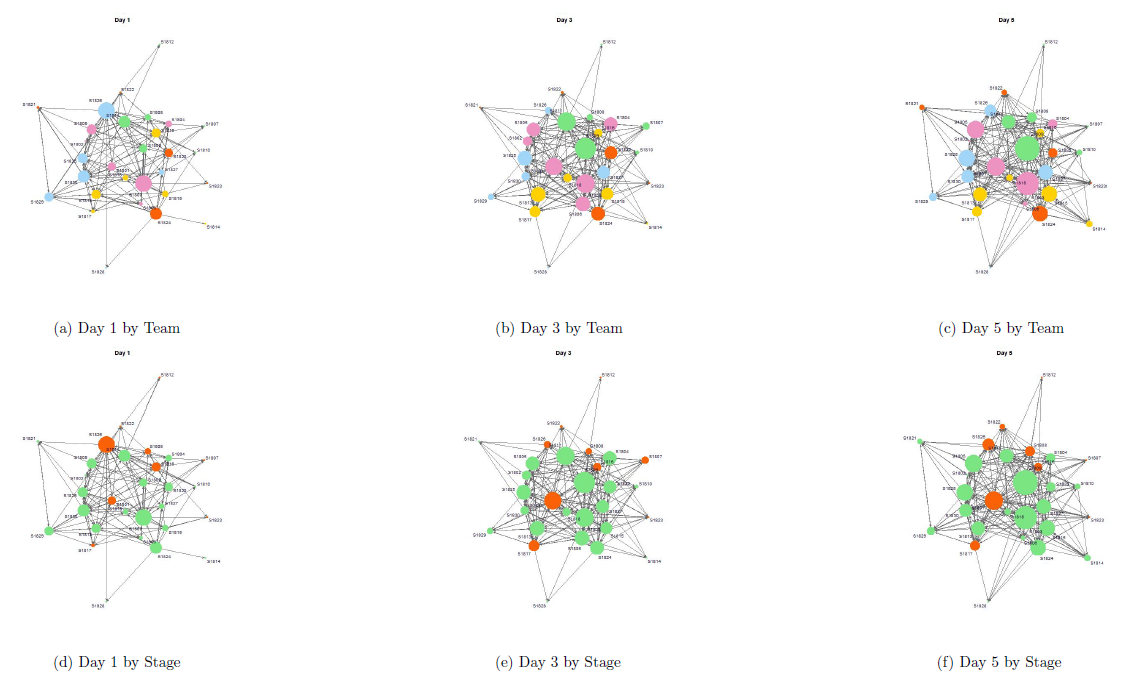** |
| --- |
| 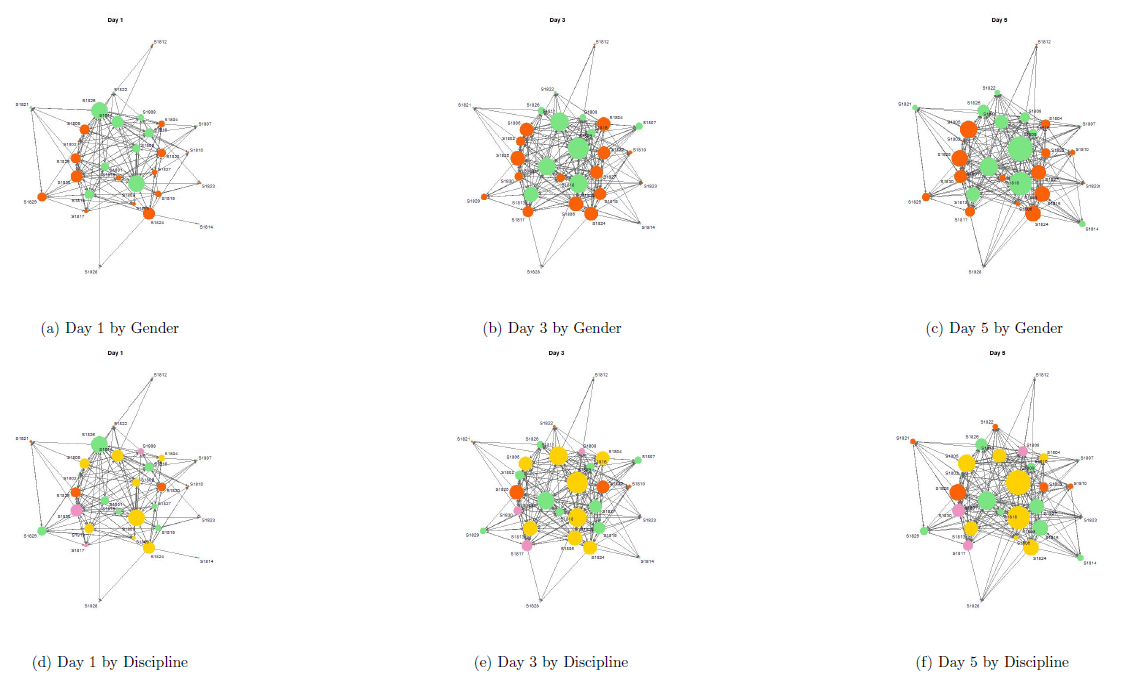 |

*Figure S4. 2019 project-based conversations per day*

| **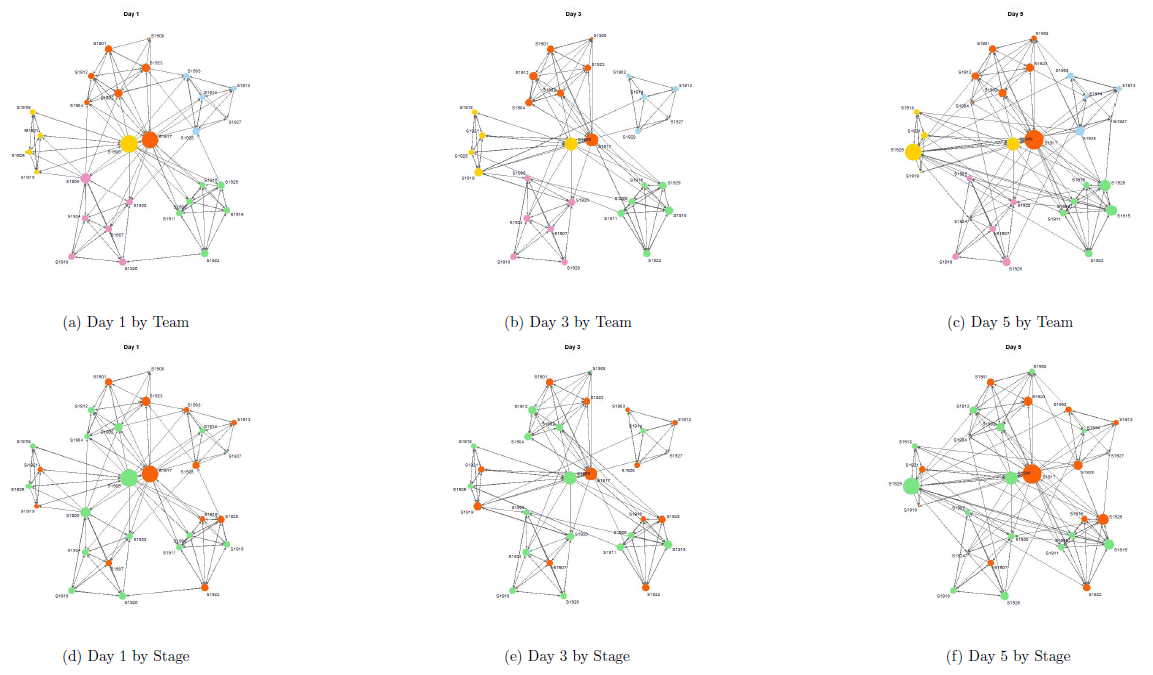** |
| --- |
| 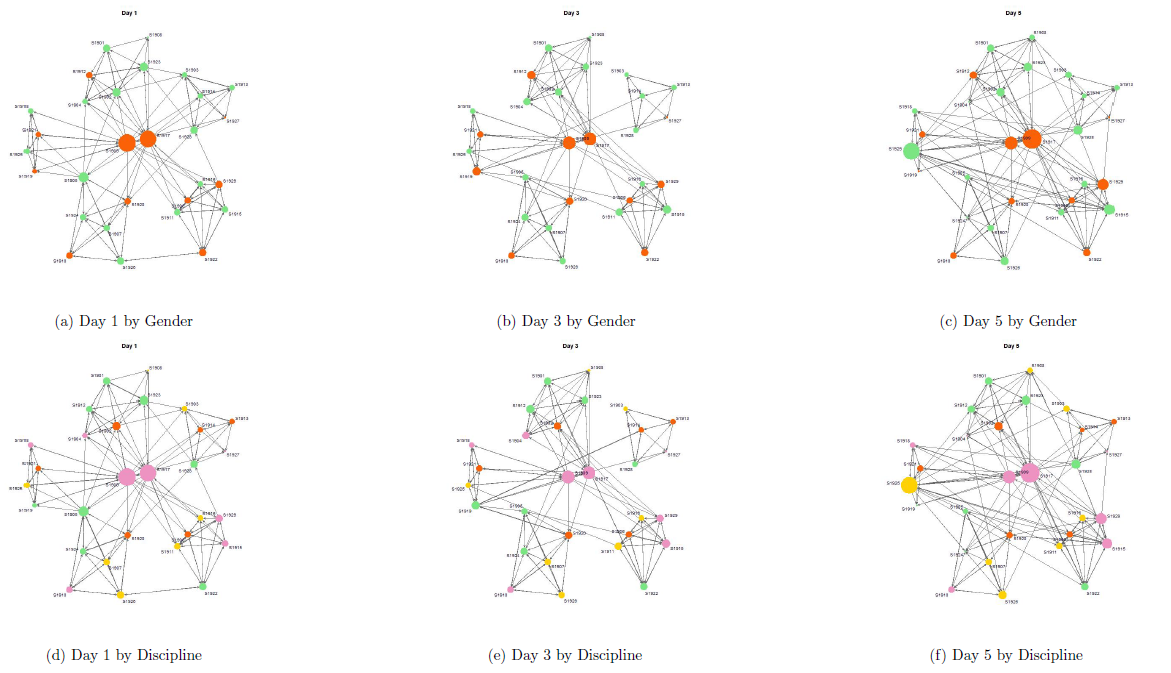 |

*Figure S5. 2019 fun conversations per day*

| **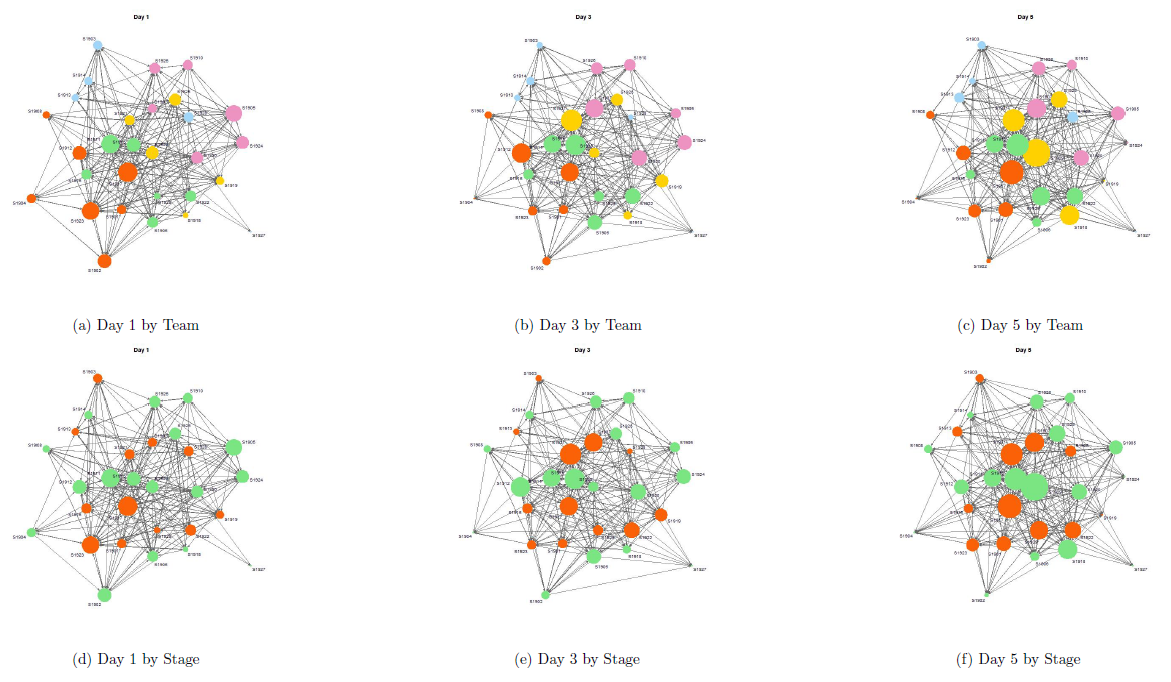** |
| --- |
| 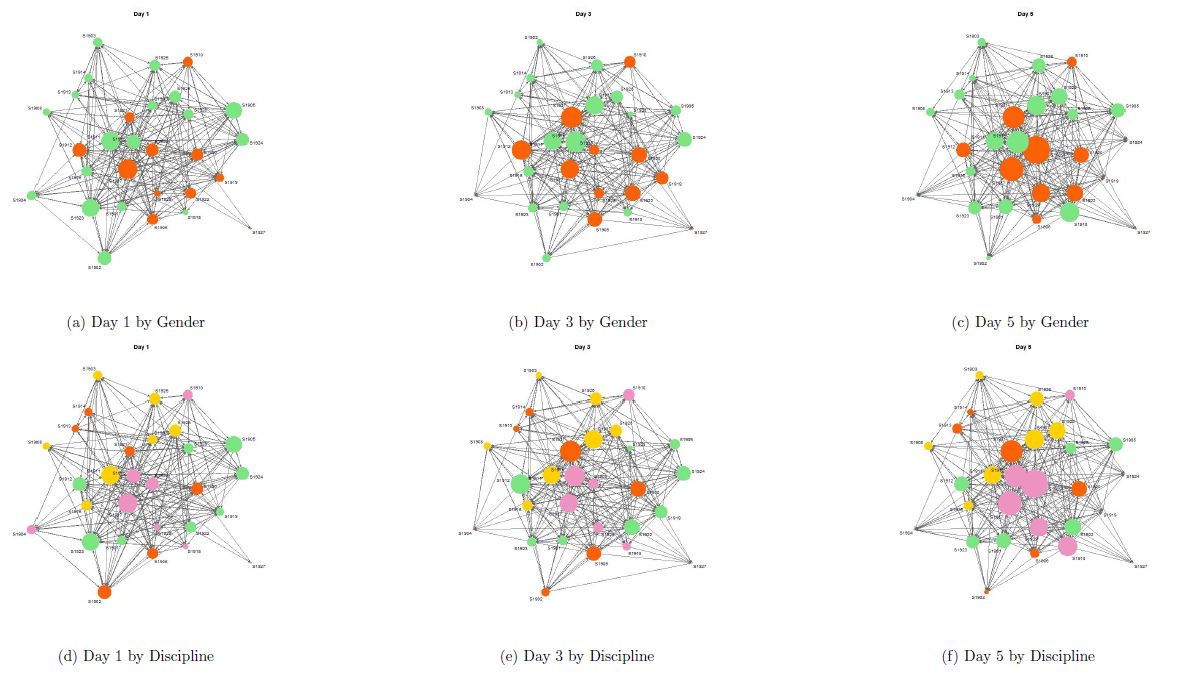 |

*S2. Convergence and goodness of fit plots*

Figures S6 to S15 show trace plots, density plots, and frequency distributions to assess the convergence and goodness of fit of Model 3 for all three years.

Convergence: The trace plots show the estimated parameters at each iteration of the Monte Carlo Markov Chains (MCMC). The density plots show the distribution of these estimated parameters. The trace plots should resemble a “hairy caterpillar” (i.e. random scatter)^1^ and the density plots should be unimodal as evidence of good mixing and convergence. These plots suggest that all fitted models were converged.

Goodness of fit: The goodness of fit of these models was assessed by simulating large numbers of networks from the estimated models and plotting the distributions of the sufficient statistics of those simulated networks (depicted as boxplots) against the sufficient statistics from the observed network (depicted as the bold dots and lines). In a good fit, the observed sufficient statistics should be close to the median of the sample sufficient statistics (e.g., outdegree and indegree).^2^ Based on this criterion, these graphs suggest that the goodness of fit of the estimated models was satisfactory.

*Figure S6. 2017 Project-based conversations (Formation)*

| 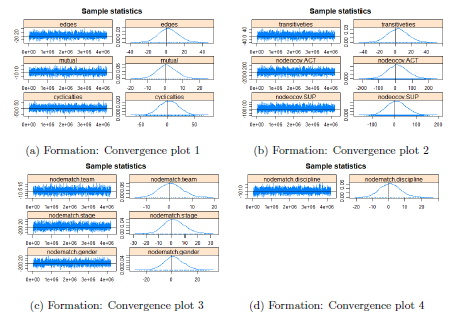  *Figure S6-1. 2017 Project-based conversations (Formation) - Convergence* | *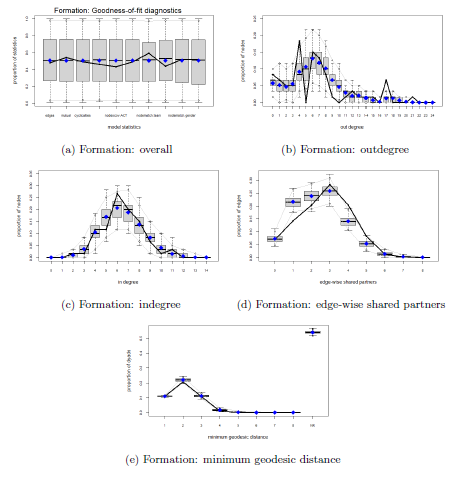*  *Figure S6-2. 2017 Project-based conversations (Formation) - Goodness of fit* |
| --- | --- |

*Figure S7. 2017 Project-based conversations (Persistence)*

| 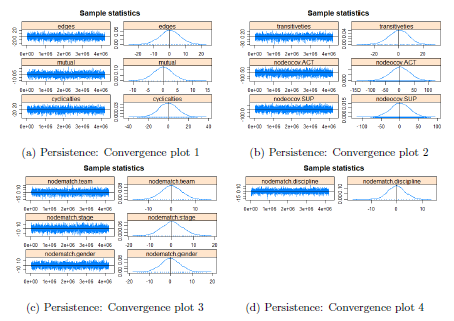  *Figure S7-1. 2017 Project-based conversations (Persistence) - Convergence* | *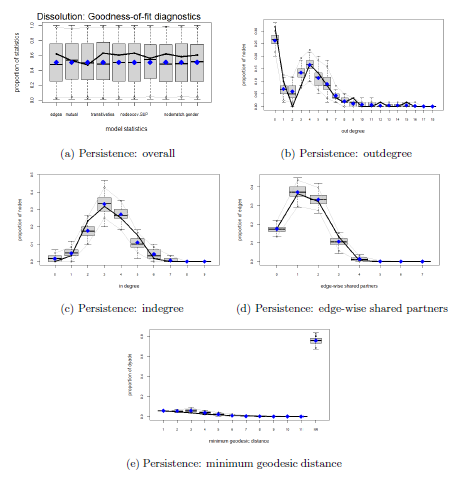*  *Figure S7-2. 2017 Project-based conversations (Persistence) - Goodness of fit* |
| --- | --- |

*Figure S8. 2018 Project-based conversations (Formation)*

| 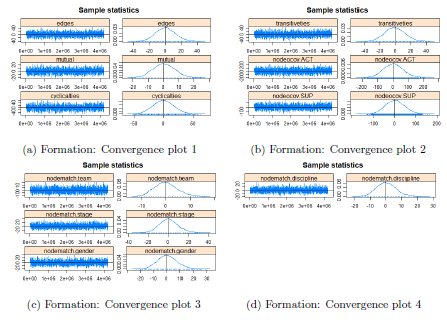  *Figure S8-1. 2018 Project-based conversations (Formation) - Convergence* | *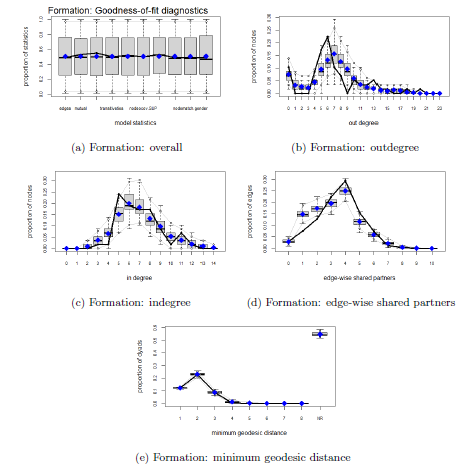*  *Figure S8-2. 2018 Project-based conversations (Formation) - Goodness of fit* |
| --- | --- |

*Figure S9. 2018 Project-based conversations (Persistence)*

| 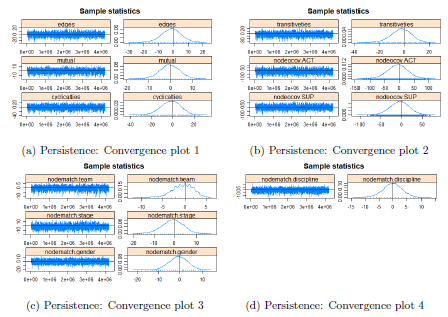  *Figure S9-1. 2018 Project-based conversations (Persistence) - Convergence* | *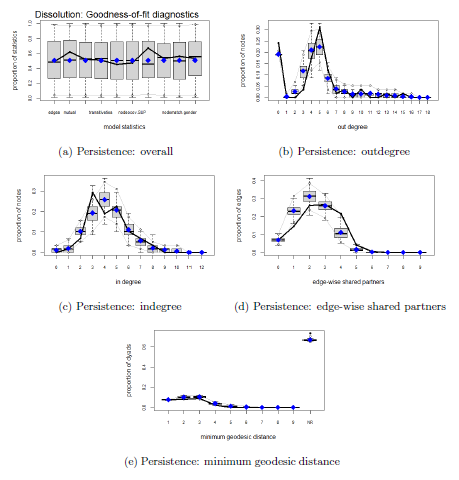*  *Figure S9-2. 2018 Project-based conversations (Persistence) - Goodness of fit* |
| --- | --- |

*Figure S10. 2018 fun conversations (Formation)*

| 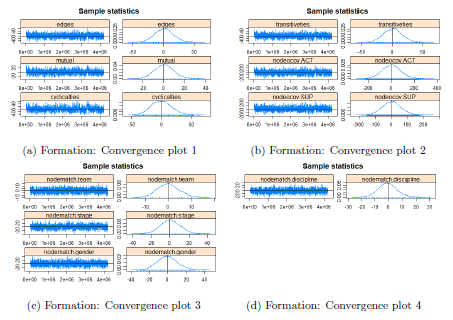  *Figure S10-1. 2018 fun conversations (Formation) - Convergence* | *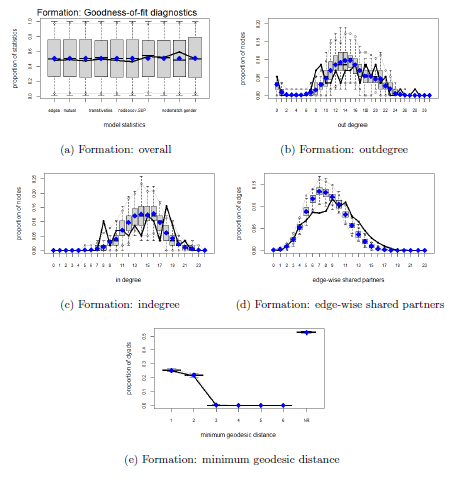*  *Figure S10-2. 2018 fun conversations (Formation) - Goodness of fit* |
| --- | --- |

*Figure S11. 2018 fun conversations (Persistence)*

| 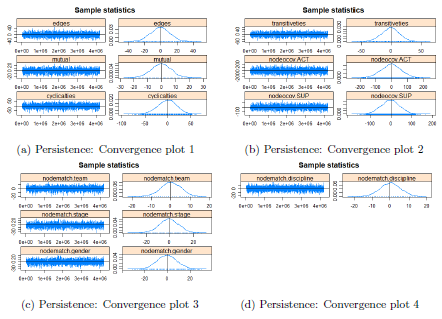  *Figure S11-1. 2018 fun conversations (Persistence) - Convergence* | *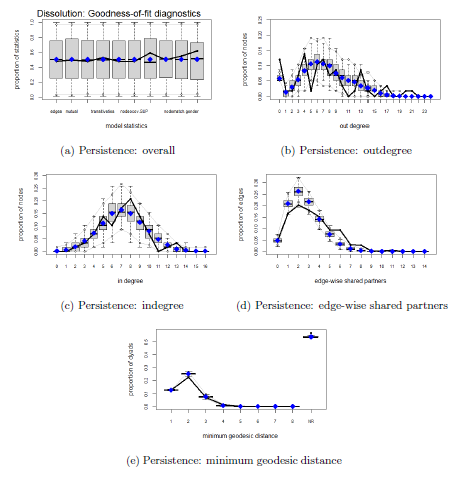*  *Figure S11-2. 2018 fun conversations (Persistence) - Goodness of fit* |
| --- | --- |

*Figure S12. 2019 Project-based conversations (Formation)*

| 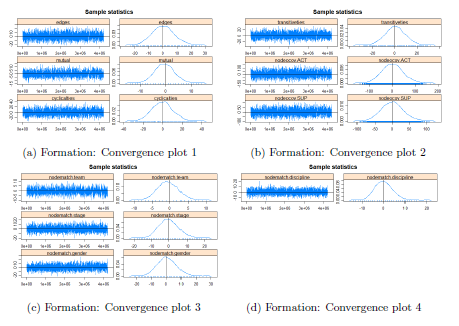  *Figure S12-1. 2019 Project-based conversations (Formation) - Convergence* | *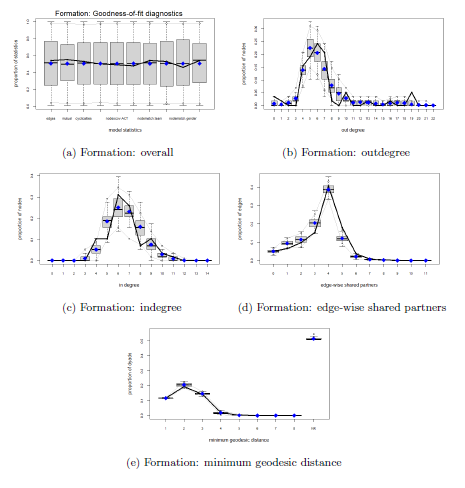*  *Figure S12-2. 2019 Project-based conversations (Formation) - Goodness of fit* |
| --- | --- |

*Figure S13. 2019 Project-based conversations (Persistence)*

| 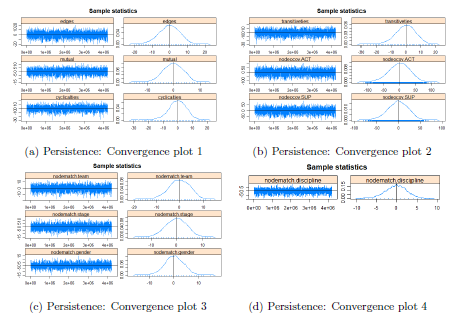  *Figure S13-1. 2019 Project-based conversations (Persistence) - Convergence* | *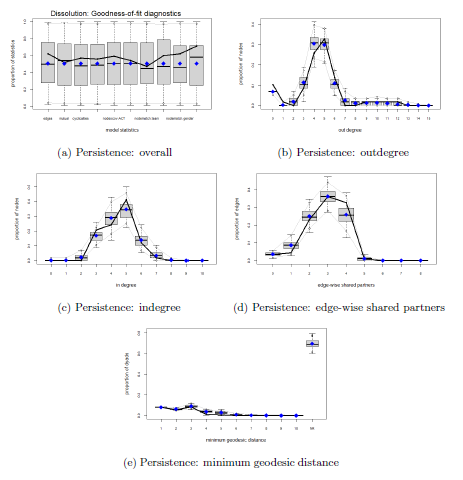*  *Figure S13-2. 2019 Project-based conversations (Persistence) - Goodness of fit* |
| --- | --- |

*Figure S14. 2019 fun conversations (Formation)*

| 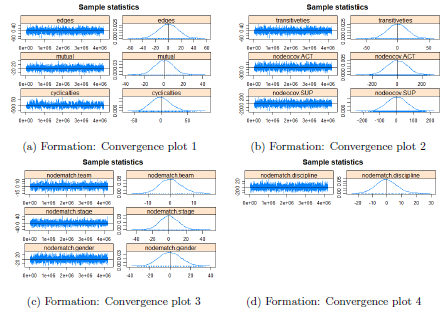  *Figure S14-1. 2019 fun conversations (Formation) - Convergence* | *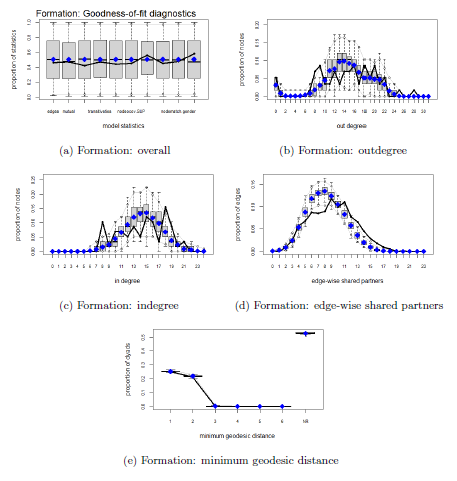*  *Figure S14-2. 2019 fun conversations (Formation) - Goodness of fit* |
| --- | --- |

*Figure S15. 2019 fun conversations (Persistence)*

| 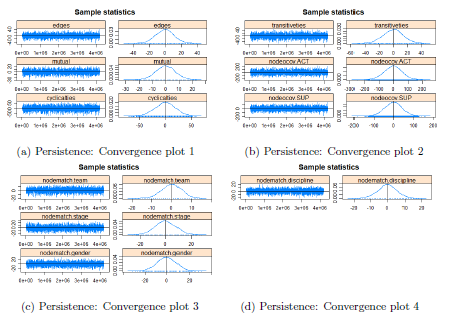  *Figure S15-1. 2019 fun conversations (Persistence) - Convergence* | *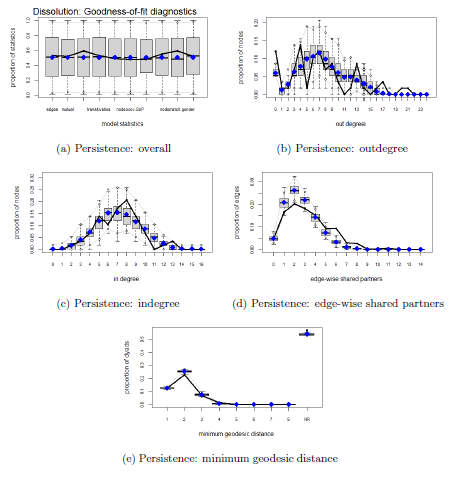*  *Figure S15-2. 2019 fun conversations (Persistence) - Goodness of fit* |
| --- | --- |

*S3. Openness to interdisciplinary collaboration items*

Here we provide additional details of the items and psychometric properties of the items used to measure “Openness to interdisciplinary collaboration”.

*1) Full item Set:* Scholars were asked to indicate the degree to which they felt they matched each of the following behaviors on a seven-point scale (from “completely false” to “completely true”).

| 1. I attend conferences outside my field |
| --- |
| 1. I read journals outside my field |
| 1. I have a readiness to collaborate with researchers outside my field |
| 1. I have a willingness to collaborate with researchers outside my field |
| 1. I obtain new insights into my own research through discussion with others outside my field. |
| 1. I have connections to fellow mHealth colleagues that have led past collaborations or that might lead to future collaborations |

2) Psychometric properties

To provide evidence on reliability and validity, we evaluated the internal consistency and the internal structure of the items by using the *psych* package in R.^3^ The evaluation was done for each year’s data as well as the merged data (of the three years’ data). The merged data was considered because the sample size of each year’s data was too small (N < 30) especially for conducting factor analysis (to provide evidence based on the internal structure). Various simulation studies suggest a minimum of N>100 for factor analysis to result in valid outcomes.^4^ The analysis with the merged data is reasonable given that it is reasonable to assume that the 2017-2019 participants came from a common population.

Reliability: We tested split-half reliability and Cronbach’s alpha. For split-half reliability, all possible splits of the test were considered. For Cronbach’s alpha, we calculated the coefficient from the full item set as well as the coefficient when each one of the items was dropped.

*Table S1. Reliability of Openness to interdisciplinary collaboration items*

| **Split-half** | | | |
| --- | --- | --- | --- |
| Average | Minimum | | Maximum |
| 0.76 | 0.58 | | 0.87 |
| **Cronbach’s alpha** | | | |
| Whole | 0.76 | Drop item 4 | 0.73 |
| Drop item 1 | 0.72 | Drop item 5 | 0.70 |
| Drop item 2 | 0.70 | Drop item 6 | 0.77 |
| Drop item 3 | 0.71 |  |  |

Validity: we tested the internal structure of the item with exploratory factor analysis. We assessed one-factor and two-factor solutions.

*Table S2. Factor analysis of Openness to interdisciplinary collaboration items*

|  | **One-factor solution** | **Two-factor solution** | |
| --- | --- | --- | --- |
| **Loadings** | Factor 1 | Factor 1 | Factor 2 |
| Item 1 | *0.44* | 0.09 | *0.62* |
| Item 2 | *0.51* | -0.04 | *0.92* |
| Item 3 | *0.81* | *1.00* | -0.02 |
| Item 4 | *0.74* | *0.68* | 0.06 |
| Item 5 | *0.59* | 0.27 | *0.43* |
| Item 6 | 0.26 | -0.05 | *0.42* |
| **Communality** | 0.35 | 0.54 | |
| **Factor correlation** | - | 1 | 0.39 |
|  |  | 0.39 | 1 |
| **p-value** | <0.001 | 0.082 | |
| **RMSEA** | 0.242 | 0.109 | |

*Note. factor loadings larger than 0.3 are italicized.

*S4. Perceived institutional support for mHealth-specific interdisciplinary collaboration items*

We provide additional details of the items and psychometric properties of the items used to measure “Perceived institutional support for mHealth-specific interdisciplinary collaboration”.

1) Full item set: Scholars were asked to indicate whether the following activities were punished, discouraged, neither encouraged nor discouraged, encouraged, or rewarded (five-point scale) by their institution.

| 1. Participation in mHealth-related professional or academic meetings outside of my primary discipline or field of study. |
| --- |
| 1. Participation in mHealth-related professional or academic meetings within my primary discipline or field of study. |
| 1. Service to mHealth-related professional groups outside of my primary discipline or field of study. |
| 1. Service to mHealth-related professional groups within my primary discipline or field of study. |
| 1. Publication on mHealth topics in journals outside of my primary discipline or field of study |
| 1. Publication on mHealth topics in journals within my primary discipline or field of study |
| 1. Collaboration on mHealth projects with researchers within my institution who come from disciplines or fields of study different from my own. |
| 1. Collaboration on mHealth projects with researchers within my institution who come from disciplines or fields of study that are the same as my own. |
| 1. Collaboration on mHealth projects with researchers outside my institution who come from disciplines or fields of study different from my own. |
| 1. Collaboration on mHealth projects with researchers outside my institution who come from disciplines or fields of study that are the same as my own. |
| 1. Development of mHealth-related intellectual property for which copyrights or patents are held by researchers at my own institution only. |
| 1. Development of mHealth-related intellectual property for which copyrights or patents are held by researchers at multiple institutions. |
| 1. Submission of mHealth-related grant proposals involving partners at other institutions. |
| 1. Submission of mHealth-related grant proposals involving partners within my institution only. |
| 1. Publication of mHealth-related journal articles in which I am the only author. |
| 1. Publication of mHealth-related journal articles in which I am the first or last author, with many co-authors. |
| 1. Publication of journal articles in which I am neither the first nor last author and am one of many co-authors. |
| 1. Development of mHealth-related software or devices that may be patented. |

To provide evidence on reliability and validity, we evaluated the internal consistency and the internal structure of the items by using the *psych* package in R.^3^ The evaluation was done for each year’s data as well as the merged data for the same reasons described above.

Reliability: We tested split-half reliability and Cronbach’s alpha. For split-half reliability, all possible splits of the test were considered. For Cronbach’s alpha, we calculated the coefficient from the full item set as well as the coefficient when each one of the items was dropped.

*Table S3. Reliability of Perceived institutional support for mHealth-specific interdisciplinary collaboration items*

| **Split-half** | | | |
| --- | --- | --- | --- |
| Average | Minimum | | Maximum |
| 0.91 | 0.79 | | 0.97 |
| **Cronbach’s alpha** | | | |
| Whole | 0.91 | Drop item 10 | 0.90 |
| Drop item 1 | 0.91 | Drop item 11 | 0.91 |
| Drop item 2 | 0.90 | Drop item 12 | 0.91 |
| Drop item 3 | 0.90 | Drop item 13 | 0.90 |
| Drop item 4 | 0.90 | Drop item 14 | 0.90 |
| Drop item 5 | 0.90 | Drop item 15 | 0.91 |
| Drop item 6 | 0.90 | Drop item 16 | 0.90 |
| Drop item 7 | 0.90 | Drop item 17 | 0.91 |
| Drop item 8 | 0.90 | Drop item 18 | 0.91 |
| Drop item 9 | 0.90 |  |  |

Validity: we tested the internal structure of the item with exploratory factor analysis. We assessed one-factor and two-factor solutions.

*Table S4. Factor analysis of Perceived institutional support for mHealth-specific interdisciplinary collaboration items*

|  | **One-factor solution** | **Two-factor solution** | |
| --- | --- | --- | --- |
| **Loadings** | Factor 1 | Factor 1 | Factor 2 |
| Item 1 | *0.49* | *0.49* | 0.05 |
| Item 2 | *0.69* | *0.40* | *0.37* |
| Item 3 | *0.48* | *0.36* | 0.14 |
| Item 4 | *0.68* | 0.18 | *0.54* |
| Item 5 | *0.65* | *0.32* | *0.41* |
| Item 6 | *0.74* | 0.04 | *0.82* |
| Item 7 | *0.65* | *0.58* | 0.16 |
| Item 8 | *0.75* | *0.43* | *0.41* |
| Item 9 | *0.76* | *1.02* | -0.11 |
| Item 10 | *0.82* | *0.84* | 0.13 |
| Item 11 | *0.32* | 0.11 | 0.17 |
| Item 12 | *0.32* | *0.38* | -0.07 |
| Item 13 | *0.76* | 0.29 | *0.55* |
| Item 14 | *0.62* | 0.07 | *0.61* |
| Item 15 | *0.30* | 0.00 | *0.35* |
| Item 16 | *0.72* | -0.11 | *0.96* |
| Item 17 | *0.48* | -0.03 | *0.58* |
| Item 18 | *0.51* | 0.19 | *0.33* |
| **Communality** | 0.38 | 0.44 | |
| **Factor correlation** | - | 1 | 0.39 |
|  |  | 0.56 | 1 |
| **p-value** | <0.001 | <0.001 | |
| **RMSEA** | 0.178 | 0.162 | |

*Note. factor loadings larger than 0.3 are italicized.

*S5. Summary of significant terms in model 3*

To aid readers’ understanding, we provide two tables indicating the significant terms of model 3 for each year and type of conversation. Tables S5 and S6 show the summarized outputs of project-based conversations and fun conversations, respectively. In both tables, ‘+’ represents a positive significant term, and ‘−’ represents a negative significant term. Non-significant terms are left blank.

*Table S5. Project-based conversations. Statistically significant factors predicting project-based conversational ties in each year of the mHTI.*

|  | 2017 | | 2018 | | 2019 | |
| --- | --- | --- | --- | --- | --- | --- |
|  | Formation | Persistence | Formation | Persistence | Formation | Persistence |
| Mutual | + |  | + | + | + | + |
| Cyclicalities | − | − | − | − | − | − |
| Transitiveties | + | + | + | + | + | + |
| Team homophily | + | + |  | + | + | + |
| STG homophily |  |  |  |  |  |  |
| GEN homophily |  |  |  |  | + |  |
| DSC homophily | + |  |  |  |  |  |
| Openness | + | + | + |  | + |  |
| Support |  |  |  |  | + |  |

*Note: STG: stage; GEN: gender; DSC: discipline.

*Table S6. Fun conversations. Statistically significant factors predicting project-based conversational ties in each year of the mHTI.*

|  | 2017 | | 2018 | | 2019 | |
| --- | --- | --- | --- | --- | --- | --- |
|  | Formation | Persistence | Formation | Persistence | Formation | Persistence |
| Mutual |  |  | + | + | + | + |
| Cyclicalities |  |  | − | − | − | − |
| Transitiveties |  |  |  | + |  | + |
| Team homophily |  |  |  | + |  | + |
| STG homophily |  |  |  |  |  |  |
| GEN homophily |  |  |  |  |  |  |
| DSC homophily |  |  |  |  |  |  |
| Openness |  |  |  |  |  |  |
| Support |  |  | + |  | + |  |

*Note: STG: stage; GEN: gender; DSC: discipline.

*S6. R code*

The R code used to run the analyses can be found at

<https://drive.google.com/drive/folders/1jyzxBniyFgmt31D78s3t5cVJ8lWmIE71?usp=sharing>

Due to data confidentiality, the data is not publicly available but may be

requested from the authors.

*S7. mHTI application and selection process*

Information about the mHTI and application process is widely disseminated through the mHTI’s website (<https://mhti.md2k.org/>), relevant email distribution lists, NIH/NSF listservs, and announcements at relevant conferences. Each annual mHTI selects approximately 30 scholars from a pool of over 300 applicants annually, selecting a combination of early, mid, and late-career scholars. However, all applicants need to be at early stages of mHealth knowledge and expertise and interested in developing integrated, transdisciplinary mHealth solutions and applying them to their area of research. Through written responses to three essay questions, applicants articulate their interest in mHealth research in the context of their career goals and long-range research agenda, concurrent or prior background in mHealth, and provide a brief summary of the mHealth solution that they expect to advance through the acquisition of mHealth expertise. Additionally, applicants provide a letter from a local mentor in their home institution and a letter of institutional support describing resources and support available to the applicant.

The selection committee, comprised of the core program faculty and select NIH/NSF program officers, reviews assigned applications based on the candidate's academic status, prior accomplishments and current evidence of a “path” towards high-quality mHealth research, letters of recommendation/support, potential for a productive mHealth research career, and responses to the three essay questions. Because a goal of the mHTI is to develop cross-disciplinary and institutional networks, special emphasis is placed on geographic and disciplinary distribution of the scholars. Scholars assigned to a team are mostly unfamiliar with one another when they arrive at the mHTI.

**References**

1. Lynch SM. *Introduction to Applied Bayesian Statistics and Estimation for Social Scientists*. Springer; 2007.

2. Handcock MS, Hunter DR, Butts CT, et al. *ergm: Fit, Simulate and Diagnose Exponential-Family Models for Networks*.; 2020. Accessed April 7, 2021. https://CRAN.R-project.org/package=ergm

3. Revelle W. *psych: Procedures for Psychological, Psychometric, and Personality Research*.; 2021. Accessed June 15, 2021. https://CRAN.R-project.org/package=psych

4. Winter JCF de, Dodou D, Wieringa PA. Exploratory factor analysis with small sample sizes. *Multivar Behav Res*. 2009;44(2):147-181. doi:10.1080/00273170902794206
